# Motility provides specific adhesion patterns and improves *Listeria monocytogenes* invasion into human HEp-2 cells

**DOI:** 10.1371/journal.pone.0290842

**Published:** 2023-08-31

**Authors:** Mariam M. Abdulkadieva, Elena V. Sysolyatina, Elena V. Vasilieva, Veronika V. Litvinenko, Egor V. Kalinin, Vladimir G. Zhukhovitsky, Natalia V. Shevlyagina, Svetlana G. Andreevskaya, Yaroslav M. Stanishevskyi, Mikhail M. Vasiliev, Oleg F. Petrov, Svetlana A. Ermolaeva

**Affiliations:** 1 Department of Infections with Natural Foci, Gamaleya National Research Centre of Epidemiology and Microbiology, Moscow, Russia; 2 Department of Dusty Plasmas, Joint Institute of High Temperatures RAS, Moscow, Russia; 3 Institute of Biochemical Technology and Nanotechnology, People’s Friendship University RUDN, Moscow, Russia; 4 Department of Bacterial Infections, Gamaleya National Research Centre of Epidemiology and Microbiology, Moscow, Russia; 5 Russian Medical Academy of Continuing Professional Education (RMANPO), Ministry of Public Health, Moscow, Russia; Okayama University, JAPAN

## Abstract

*Listeria monocytogenes* is motile at 22°C and non-motile at 37°C. In contrast, expression of *L*. *monocytogenes* virulence factors is low at 22°C and up-regulated at 37°C. Here, we studied a character of *L*. *monocytogenes* near surface swimming (NSS) motility and its effects on adhesion patterns and invasion into epithelial cells. *L*. *monocytogenes* and its saprophytic counterpart *L*. *innocua* both grown at 22°C showed similar NSS characteristics including individual velocities, trajectory lengths, residence times, and an asymmetric distribution of velocity directions. Similar NSS patterns correlated with similar adhesion patterns. Motile bacteria, including both pathogenic and saprophytic species, showed a preference for adhering to the periphery of epithelial HEp-2 cells. In contrast, non-motile bacteria were evenly distributed across the cell surface, including areas over the nucleus. However, the uneven distribution of motile bacteria did not enhance the invasion into HEp-2 cells unless virulence factor production was up-regulated by the transient shift of the culture to 37°C. Motile *L*. *monocytogenes* grown overnight at 22°C and then shifted to 37°C for 2 h expressed invasion factors at the same level and invaded human cells up to five times more efficiently comparatively with non-motile bacteria grown overnight at 37°C. Taken together, obtained results demonstrated that (i) NSS motility and correspondent peripheral location over the cell surface did not depend on *L*. *monocytogenes* virulence traits; (ii) motility improved *L*. *monocytogenes* invasion into human HEp-2 cells within a few hours after the transition from the ambient temperature to the human body temperature.

## Introduction

Motility is a huge advantage for bacterial pathogens as it provides the ability to get favorable environments and cognitive targets [1–3]. Flagellar motility is essential for virulence of many pathogenic bacteria and particularly enteric pathogens including *Escherichia coli*, *Salmonella* Typhimurium, *Clostridium difficile*, *Helicobacter pylori*, *Campylobacter jejuni*, and *Listeria monocytogenes* [4–8]. Some enteric pathogens such as *H*. *pylori* need flagellar motility continuously during infection and cannot persist within the mammalian host without being motile and competent for chemotaxis [1, 8] Others such as *L*. *monocytogenes* and enteropathogenic *Yersinia* spp are motile when grow in the environment but lose flagella upon adaptation to the host conditions [9, 10]. However even for *L*. *monocytogenes* and pathogenic *Yersinia* that lose their flagella at latter stages of infection, chemotaxis and flagellar motility are essential for early colonization of the gastrointestinal tract [7, 11]

From the point of view of pathogenesis strategies, the role of motility in the course of pathogen interactions with the surface is of the highest importance [12–14]. Interactions of motile bacteria with the surface can be divided into a few stages: (i) landing, i.e. exit of the bacterium from the liquid bulk that is characterized by changes in flagella rotation or tumbling frequency comparatively with the behavior of bacteria within the bulk of the liquid; (ii) near-surface swimming (NSS) when short-term interactions with the surface prepare the bacterium for stopping; (iii) stopping characterized by prolonged interactions between the bacterium and the surface; (iv) docking, i.e. adhesion to the surface, or, alternatively, separation and return to the bulk in dependence on efficiency of interactions with the surface [15–18].

There are a few distinct movement modes including swimming, swarming, gliding, sliding and twitching [19]. Swimming and swarming are performed via rotation of the flagella bundle while other motility modes use other surface structures and mechanisms [14, 20–22]. Swimming is defined as motility of individual bacterial cells moving in the liquid environment that is performed via distinct patterns such as run-tumble, run-reverse or run-reverse-flick depending on the arrangement of bacterial flagella and/or other structures [23–25]. Swimming bacteria approach the surface under a relatively big impact angles and upon approaching the surface they slow down, change the position of the body and keep near surface “nose down” swimming with the pitch angle of about 10° and the flagellar bundle directed away from the surface [16]. Swarming is defined as the motility in association with other bacterial cells in a thin film of liquid over a moist surface [14, 20, 23]. Swarming bacteria keep their bodies parallel to the surface with average angle as low as 0.7° [24, 25–28]. Recently, we have demonstrated that *E*. *coli* strains with distinct modes of the landing and NSS motility demonstrate different adhesion patterns to the surface of epithelial cells [29]. The *E*. *coli* strain moving in the parallel to the surface “horizontal” mode accumulated along the cell-cell border of the epithelial cell monolayer while the strain moving with high impact angles evenly distributed over the cell surface [29]. To support these observations and investigate an impact of motility-driven bacterial distribution over surface on virulence, we applied developed methods to analyze motility and adhesion patterns of another enteric pathogen, *L*. *monocytogenes*.

The food-borne human pathogen *L*. *monocytogenes* is a facultative intracellular parasite that enters the body with contaminated food, crosses the intestinal barrier and colonizes internal organs including the liver, spleen, brain, placenta, and fetus by exploiting an ability to invade and multiply in nonprofessional phagocytes [30]. *L*. *monocytogenes* invasion into epithelial cells and other nonprofessional phagocytes requires products of the *inlAB* locus, the internalin-family surface proteins InlA and InlB [31]. The *inlAB* operon is a part of the *L*. *monocytogenes* virulence regulon and its expression is dependent on growth temperature activating at the temperature of the human body of 37°C [32].

*L*. *monocytogenes* has four to six flagella per cell that move in a manner similar to *E*. *coli* and other peritrichous bacteria [33]. Activity of the *L*. *monocytogenes* flagellar genes is temperature dependent, and the bacterium is motile at temperatures below 30°C but is not flagellated and non-motile at 37°C [10, 33]. Thus, the switch from ambient to physiological temperature downregulates flagella and upregulates the *L*. *monocytogenes* virulence regulon [10, 34]. Still, flagellar motility was shown to be essential for *L*. *monocytogenes* epithelial cell invasion and intestine infection [7, 35–37].

Besides *L*. *monocytogenes*, the genus *Listeria* includes 20 species, which are all but one saprophytic, i.e. obtaining nourishment from the products of organic breakdown and decay [38]. The saprophyte species *L*. *innocua* is the closest to *L*. *monocytogenes* species in terms of the genetic distance [39]. In contrast to *L*. *monocytogenes*, *L*. *innocua* is flagellated and motile at both ambient and human body temperatures [40].

Marquis and O’Neil demonstrated that it is motility rather than flagella themselves that improves *L*. *monocytogenes* invasion into epithelial cells [7]. To understand how motility affects *L*. *monocytogenes* interactions with the host cell and to establish correlations between motility and adhesion patterns, we analyzed *Listeria* spp motility at the NSS stage and corresponding adhesion patterns in dependence on the surface type. Further, we evaluated effects of motility on efficiency of *L*. *monocytogenes* invasion into human cells in dependence on invasion factor production.

## Materials and methods

### Bacterial strains and cultivation conditions

The *L*. *monocytogenes* strain EGDe [39] and the *L*. *innocua* strain SLCC 3379 (serovar 6a) were used. Bacteria routinely cultivated on the Brain Heart Infusion (BHI, BD, USA) medium at 22°C. For the experiment, the isolated colony from the fresh plate was seeded into the BHI broth and grown at 22°C or 37°C with shaking at 180 rpm min^-1^ overnight.

### Cell culture

The Human Epithelial type 2 (HEp-2) cells derived from larynx adenocarcinoma were cultivated in the DMEM (Paneko, Russia) medium supplemented with 10% (vol/vol) fetal bovine serum (Gibco, USA) without antibiotics at 37°C in an atmosphere of 5% CO_2_.

### NSS motility observation and characterization

The observation and characterization of *Listeria* spp motility was generally performed as described earlier [29]. Bacteria were grown in the BHI broth at 22°C or 37°C overnight. Then the culture was diluted with fresh BHI (1:9 culture and BHI, respectively) and incubated at the same temperature for 7 h. When the effect of the temperature shift was analyzed, overnight culture was diluted and then incubated at 22°C for 5 h. After that the obtained bacterial suspension was separated into two samples, one of which was kept under the same conditions, while another was moved to 37°C, and samples were grown for 2 h more for the total time of incubation to be 7 h.

For NSS motility observation, the culture grown for 7 h was diluted up to the optical density OD_600_ = 2.0 that corresponded to the cell density of 1x10^9^ cfu ml^-1^ and the diluted culture was placed into the PLA microfluidic chamber with a straightforward channel 30 μm deep and 10 mm wide. A glass coverslip was rubbed to the surface of the chamber. A series of 30 second video images of moving bacteria were recorded at a speed of 30 frames per second by a bright field microscopy with the Zeiss Axio Scope A1 microscope (objective Achroplan 40x/0.65) equipped with a digital camera DCM510 with CMOS chip 5Mpx (China) that provided the resolution up to 2560 x 1920 px. At least 5 videos were recorded for each sample, all experiments were repeated at least 3 times. The observation was carried out at the vicinity of the channel bottom. Approximately 10 seconds of the video stream were cut for video processing and analysis. The videos were processed using the Plasma 4.0 software, which was developed to analyze active microparticles [41]. The Plasma 4.0 software provided a statistical subtraction of the background, Fourier filtering of the image, frame-by-frame identification of the location of each of the bacteria to obtain coordinates of the bacteria, their trajectories, velocities, and mean-square displacements <Δr2(t)>. We took into account trajectories longer than 10 frames to eliminate false detections, specifically the blurry circles with short trajectories less than 10 frames that present throughout the preprocessing stage.

### Quantification of bacterial adhesion to the plastic surface

2 ml of the overnight culture grown and diluted up to OD_600_ = 2.0 as described above were added to wells of the 24-well plates (Thermo Fisher Scientific Inc, cat. no. 142475) and incubated at the same temperature, at which the culture had been grown. After 15 or 60-minute incubation, the liquid medium and non-adsorbed bacteria were removed, the wells were washed three times with sterile phosphate-buffered saline (PBS) by gentle adding, manual swirling and removing of the medium. The adhered bacteria were scraped with a sterile cotton swab and resuspended in 2 ml PBS. Decimal dilutions of the suspension were plated on the BHI agar; plates were incubated at 37°C to count bacterial colonies 24 h later. The efficiency of adhesion was calculated as a percentage of adhered bacteria to the number of bacteria used to inoculate the well.

### Visualization of bacterial adhesion to the plastic surface by light microscopy

To analyze patterns of bacterial adhesion to the plastic surface, a 1 cm^2^ piece of plastic was put on the bottom of the well of the 24-well plate and the culture was added and processed as described above. After gentle washing, bacteria were fixed with 3.7% formaldehyde (pH 7.2) for 10 minutes, washed again three times with sterile PBS, and stained with Hoechst 33342 (Thermo Fisher Scientific, USA) according to the manufacturer’s instructions. Bacteria were visualized using epifluorescence microscopy at a 1000-fold magnification using a Zeiss Axio Scope A1 microscope (objective Achroplan 100x/1.25 oil ph3) equipped with a DCM510 digital camera.

### Quantification of bacterial adhesion to the surface of HEp-2 cells and characterization of adhesion patterns by light microscopy

HEp-2 cells were grown in 24-wells plates up to 70% confluence. Then 1 ml of the overnight culture grown at the temperature pointed in the text with the OD_600_ = 2.0 was added to the cell monolayer. After15 or 60 minutes, bacterial suspension was removed and cells were thoroughly washed with PBS three times as described above. Then, 100 μL 1% TritonX-100 was added, cells were incubated for 15 seconds before 900 μL PBS was added. Decimal dilutions of cell lysates were plated to count the number of cell-bound bacteria as described above. Visualization of the adhesion patterns was performed after fixation of the cell monolayer with adhered bacteria with formaldehyde and staining with Hoechst 33342 as described above for bacteria adhered to the plastic surface.

### Quantification of bacterial positioning over the cell surface

The relative distance of the adhered bacterium from the nucleus was determined using the light microphotographs as shown at the S1 Fig. Processing of images was performed as follows: 1) we identified the position of the bacteria and cell borders; 2) radial lines were drawn from the nominal cell center through the bacterium to the cell border; 3) the line lengths from the nucleus edge to the cell edge were divided into deciles and bacterial positions were prescribed to the particular decile. All bacteria over the cell nucleus were prescribed to the first decile. Totally 10 microphotos taken with a Zeiss Axio Scope A1 microscope equipped with a DCM510 digital camera at a 1000-fold magnification were analyzed for each sample.

### Scanning electron microscopy

For SEM, cell cultures with adhered bacteria were washed and fixed as described above. SEM was performed using the Camscan S2 microscope (Cambridge Scientific Instruments, Witchford, UK) in the secondary electron imaging (SEI) mode with a 10 nm optical resolution and an operating voltage of 20 kV. The images were captured using the MicroCapture software (SMA, Moscow, Russia).

### Cell invasion

Bacteria were prepared as for the adhesion assay and 1 ml was added to 70% HEp-2 cell monolayer at the OD_600_ = 2 that corresponded to MOI (multiplicity of infection) ≈ 100: 1 (bacteria: cells). Bacteria were incubated with cells for 15 or 60 minutes, then the bacterial suspension was removed, cells were washed with PBS three times and the fresh DMEM medium supplemented with 100 μg ml^-1^ gentamicin was added to remove extracellular bacteria. After 1 h incubation, the medium was removed, cells were gently washed with PBS three times, lysed with TritonX-100 as described above and decimal dilutions of cell lysates were plated on BHI agar to count colonies formed by intracellular bacteria. The efficiency of invasion was evaluated as the percentage of intracellular bacteria to the total number of bacteria added to the well.

### ELISA

To evaluate amounts of surface-bound InlB, 100 μl overnight bacterial culture grown under conditions described in the text were added to wells of a 96-well plate. The plates were incubated at 37°C overnight, washed three times with 250 μl of TTBS (Tris-buffered saline supplemented with 0.05% Tween-20), and then free bounding sites were blocked by adding 200 μl 2% bovine serum albumin (BSA) dissolved in TTBS. After 30 minutes incubation, the blocking buffer was removed and 100 μl conjugate of the rabbit polyclonal InlB specific antibodies with horseradish peroxidase (anti-InlB-HRP; [42]) diluted 1:4000 in the blocking buffer was added. Bacteria were incubated with anti-InlB-HRP at room temperature with shaking at 140 rpm for 1 hour. Then, wells were washed with TTBS 6 times and HRP enzymatic activity was revealed using 100 μl TMB substrate (Bio-Rad). To cease the reaction, 100 μl 2 M sulfuric acid was added. The optical density was measured at a wavelength of 450 nm using the iMark plate photometer (Bio-Rad). InlB amounts were detected relatively to the calibration curve that was developed using the purified protein [42].

### Statistics

All experiments were repeated at least three times. The mean and standard deviation (SD) values were calculated from the entire data set where applicable. Statistical analysis was performed using one-way ANOVA with the post hoc Tukey’s test. The homogeneity of variance assumption was tested using Levene’s test. Statistical differences were considered significant when the p-value was <0.05.

## Results

### *L*. *monocytogenes* and *L*. *innocua* demonstrated similar velocity profiles at the NSS stage

Here, we compared motility patterns of two *Listeria* species: the pathogenic species *L*. *monocytogenes* strain EGDe (further designated as Lm) and the saprophytic species *L*. *innocua* strain SLCC 3379 (further designated as Linn). Linn was grown at 22°C. Lm was grown at two temperatures, 22°C and 37°C. Lm grown at 22°C was motile, while Lm grown at 37°C was non-motile (further designated as non-motile Lm) that is in agreement with temperature-dependent regulation of *L*. *monocytogenes* motility [10, 33, 34, 40].

**Fig 1:**
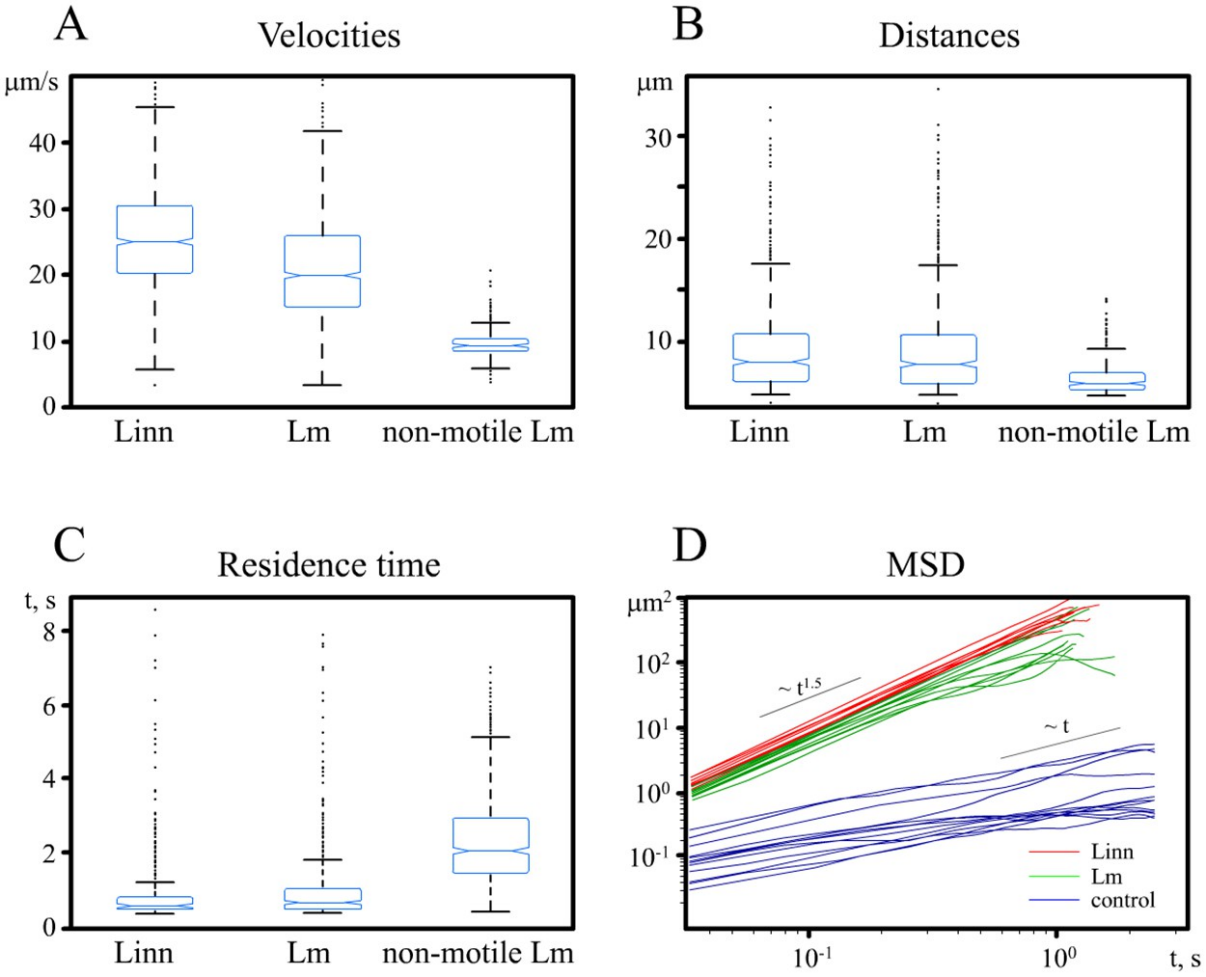
NSS characteristics. (A)–bacterial velocities, (B)–characteristic distances, representing lengths of bacterial trajectories, (C)–residence time; (D)–time dependences of mean square displacements for selected bacteria. Motile Lm–*L*. *monocytogenes* grown at 22°C; non-motile Lm–*L*. *monocytogenes* grown at 37°C; Linn–*L*. *innocua* grown at 22°C.

Bacterial motility was followed at the NSS stage, which is the last and the most important stage before stopping and surface adhesion (docking) take place. The data sets used in the calculations included 10 independent observational areas video-recorded for 10 seconds. Experimental conditions allowed monitoring of 450–500 bacteria moving within the observation plane during 10 seconds. The concentration of bacteria simultaneously in the field of view ranged 1–2 х 10^−3^ bacteria per μm^2^. Main characteristics of bacterial motility are shown in Fig 1, including individual instantaneous velocities (Fig 1A), characteristic distances, representing lengths of bacterial paths, which were calculated as major diagonals of polygons circumscribed around bacterial trajectories (Fig 1B), residence time, i.e. a time that an individual bacterium stayed within the horizontal layer (microscope field of view) before leaving (Fig 1C) and mean square displacements (MSD) on time (*t*) (Fig 1D).

The median velocities of motile bacteria were 20 μm/s (CI 95 19.6–20.4) and 25 μm/s (CI 95 24.8–25.5) for Lm and Linn, respectively that was more than two-times higher than the median velocity of taken as a control of Brownian movement non-motile Lm, which was 9 μm/s (CI95 8.8–9.2) (p<0.01) (Fig 1A). The median residence times demonstrated by motile Lm and Linn were similar (0.62 s (CI 95 0.6–0.65); and 0.53s (CI 95 0.52–0.54), respectively) and more than three-times shorter than the residence time of non-motile Lm (2 s (CI95 1.9–2.1); p<0.01). The median distances, i.e. the distance that individual bacteria ran within the layer before leaving, were slightly longer for motile than non-motile bacteria (8 μm (CI 95 7.8–8.2); 8.1 μm (CI 95 8–8.4); and 6 μm (CI 95 5.9–6.1) for Lm, Linn, and non-motile Lm, respectively; p<0.01). Individual trajectories of motile bacteria were relatively linear compared to the individual trajectory of non-motile Lm (S1 Fig). Taken together, these data demonstrated that average parameters of motility were similar for motile bacteria of pathogenic and saprophytic species and were distinct for motile and non-motile bacteria.

**Fig 2:**
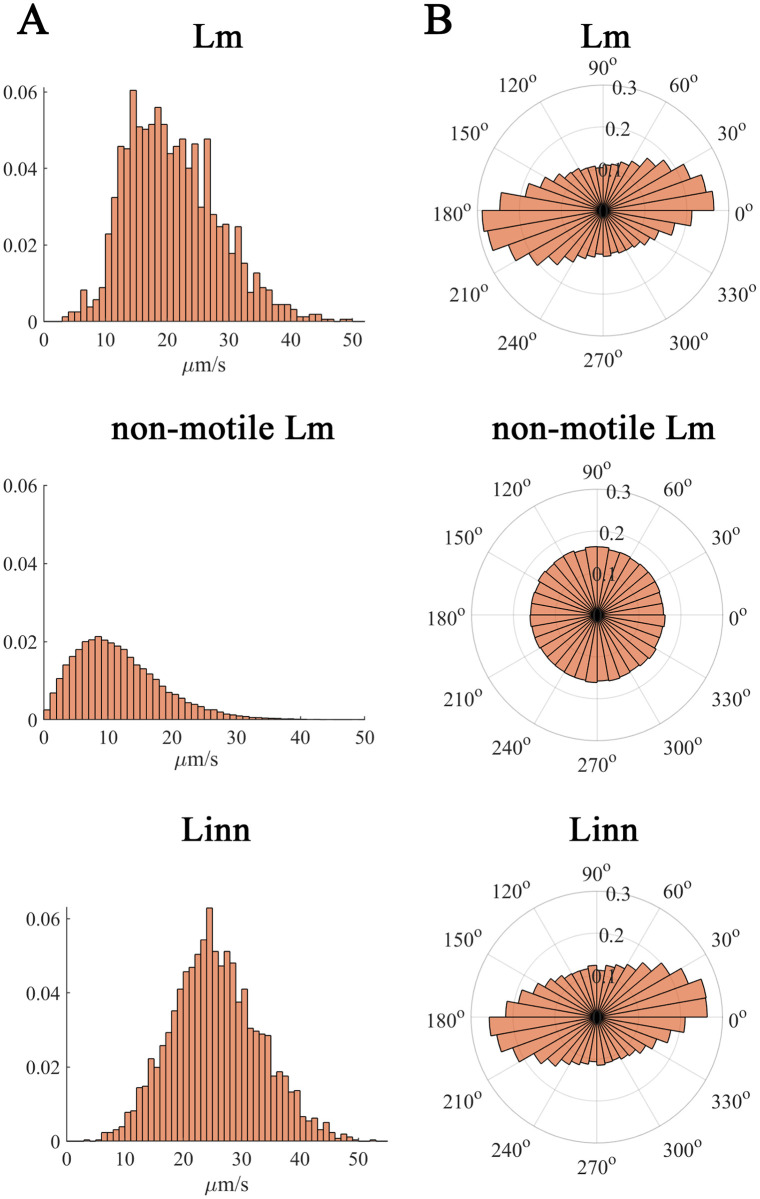
Instantaneous velocity distribution by module (A) and direction (B).

The velocity profile distribution for motile bacteria has a normal (Gaussian) character (Linn) or was close to a normal distribution (Lm) (Fig 2A). Velocity profile of non-motile Lm deviated from the normal distribution due to a noticeable prevalence of bacteria with velocities from zero to 7 μm s^-1^ that is in line with the absence of active motility. The distribution of instantaneous velocity directions was symmetric for non-motile Lm and asymmetric for motile bacteria (Fig 2B). Such an asymmetry suggested a coordinated motility of the individual cells [43, 44].

To further characterize bacterial motility and to support the suggestion about the predominance of the directed type of motility over a chaotic component of the motion, we calculated the dependence of the mean square displacement (MSD) on time (*t*). For the non-motile Lm culture, a typical for the classical Brownian dynamics linear MSD time-dependence was found (Fig 1D). For motile Lm and Linn displacement MSD was proportional to the time taken to the power 1.5 (~ t^1.5^) that points to active movement in horizontal plane.

Taken together, obtained results indicate that pathogenic and saprophytic *Listeria* spp exhibit similar motility patterns, which are distinct from the patterns demonstrated by the control culture of non-motile Lm.

### Motile *Listeria* spp demonstrated similar adhesion patterns while adhesion efficiencies were individual

**Fig 3:**
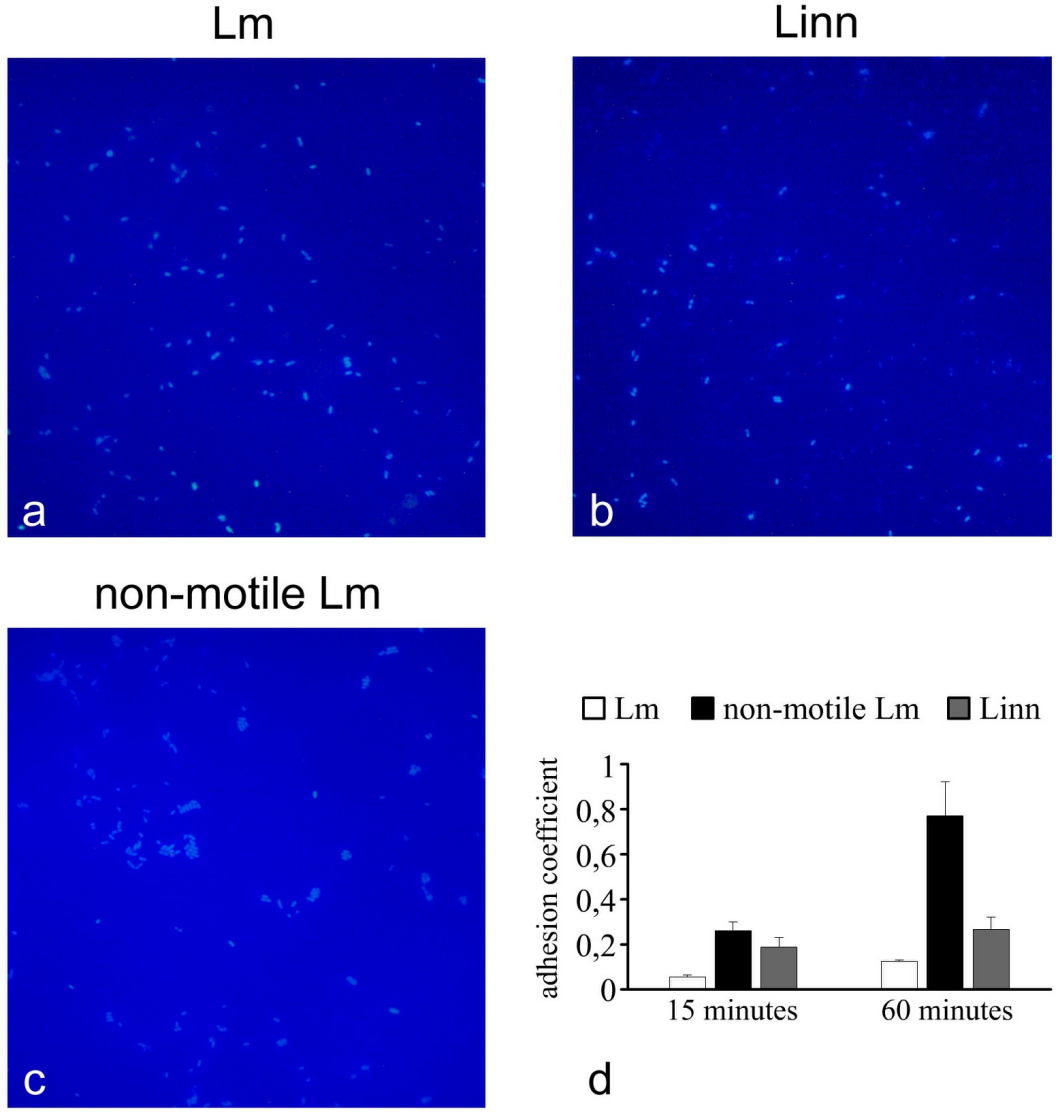
Adhesion to the plastic surface. Illustration of typical patterns of bacterial adhesion for *L*. *monocytogenes* (a), *L*. *innocua* (b), and non-motile *L*. *monocytogenes* (c), respectively. (d)–efficiency of adhesion.

Using the light microscopy, we analyzed adhesion patterns of non-motile and motile *Listeria* spp at early stages of interactions with the surface, 15 and 60 minutes after bacterial suspension was applied to a 24 well plate (Fig 3). After 15 minutes, motile bacteria of both species adhered primarily as randomly distributed single cells. Adhesion patterns of motile pathogenic and saprophytic *Listeria* spp were very similar after both 15 and 60 minutes suggesting that motility driven adhesion patterns do not depend on virulence traits. Non-motile bacteria were often observed as small aggregates including up to 10 cells. The plastic surface and short observation time did not allow cell division, so these groups could result rather from sedimentation of floccules formed within the liquid bulk. Taking into account all the aforementioned points, it might be suggested that motility prevented bacterial flocculation within the liquid bulk.

To quantify adhesion efficiencies, adhesion coefficients, i.e. the ratio of bacteria adhered to the plastic surface to bacteria added to the well, were calculated (Fig 3D). 15 minutes post addition, the adhesion coefficient of motile Lm was at least 4.5 times less than non-motile Lm (p<0.05). Adhesion efficiency of Linn and non-motile Lm differed insignificantly (p = 0.42.). Upon prolongation of the incubation up to 60 minutes, the adhesion coefficients increased for all bacteria tested with the highest increment observed for non-motile Lm. The difference between non-motile and motile bacteria increased up to 6.2- and 2.9- times for motile Lm and Linn, respectively (p<0.05 for all). Thus, motile *Listeria* spp demonstrated similar adhesion patterns especially at the early stage of observation meanwhile adhesion efficiencies were individual.

### Motility determined *Listeria* spp positioning at the surface of the HEp-2 cells

**Fig 4:**
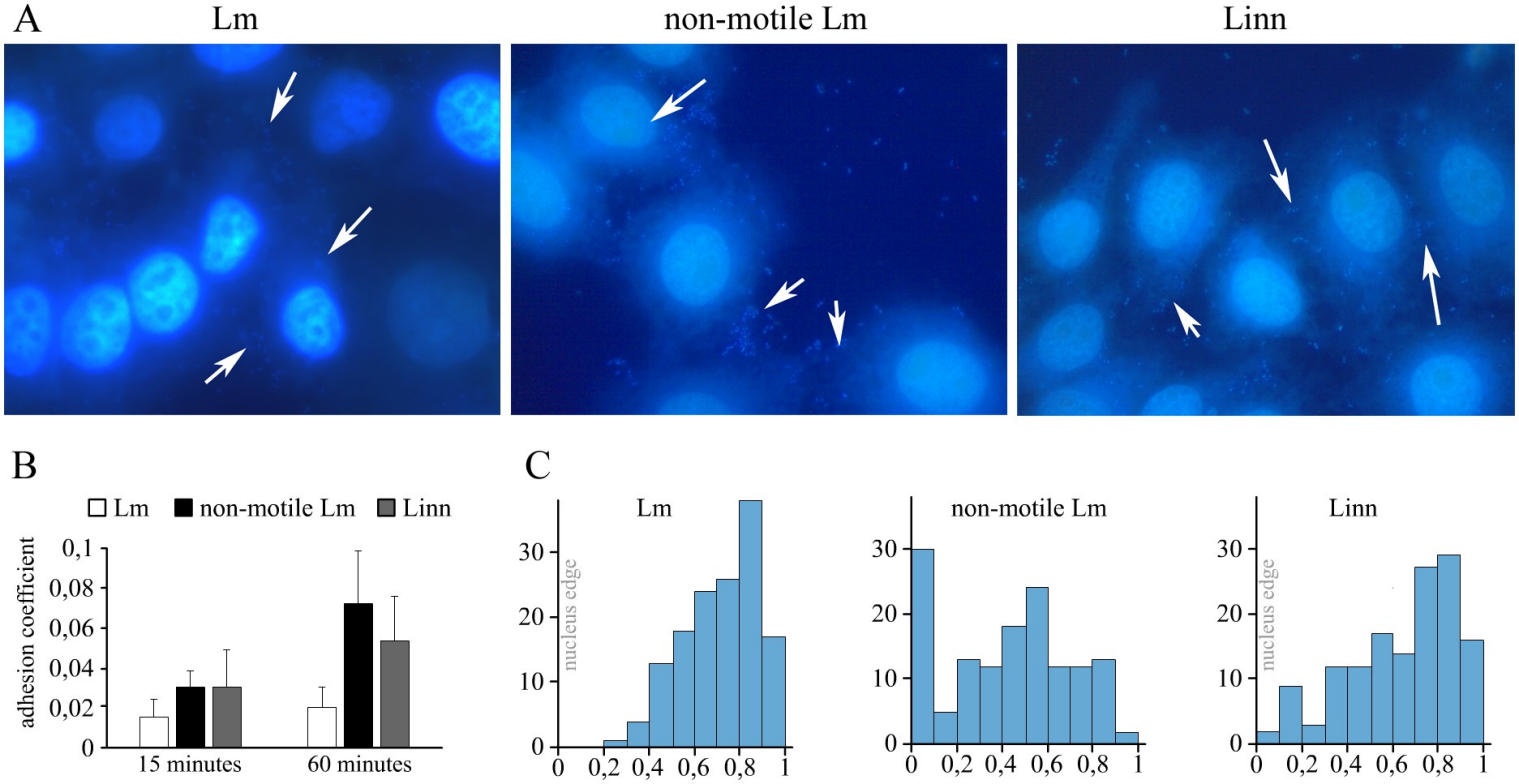
Adhesion to the surface of epithelial cells HEp-2. (A)–adhesion patterns of Lm, non-motile Lm and Linn over the HEp-2 epithelial cell monolayer 60 minutes post bacterial addition to the cell monolayer; white arrows point bacterial cell positions. (B)–adhesion efficiencies showed as a percentage of adhered bacteria to the number of added bacteria; (C)–distribution of bacteria relatively to the cell nucleus (see Materials and methods).

Next question we addressed was patterns of bacterial adhesion to the surface of human cervical adenocarcinoma epithelial HEp-2 cells. Non-motile Lm were distributed randomly over the cell monolayer surface including area over nuclei (Fig 4A). Formless aggregates were observed similar to aggregates on the plastic surface suggesting the same gravity-stimulated sedimentation of flocs formed by non-motile bacteria within the liquid bulk. In contrast, both motile Lm and Linn were predominantly located at the cell periphery. The difference in the number of Lm and Linn adhering to the cell surface was less pronounced than on the plastic surface (Fig 4B). To confirm the visual impression of an uneven distribution of bacteria, the bacterial distribution relative to the cell nucleus was calculated (see Materials and methods and S2 Fig). The quantitative data supported the view on the uneven bacterial distribution. Particularly, a part of non-motile bacteria settled down over the nucleus and/or directly next to the nucleus (Fig 4C). The number of motile bacteria in the direct vicinity of the nucleus was negligible. Percentage of bacteria at cell periphery was higher for motile bacteria comparatively to non-motile.

**Fig 5:**
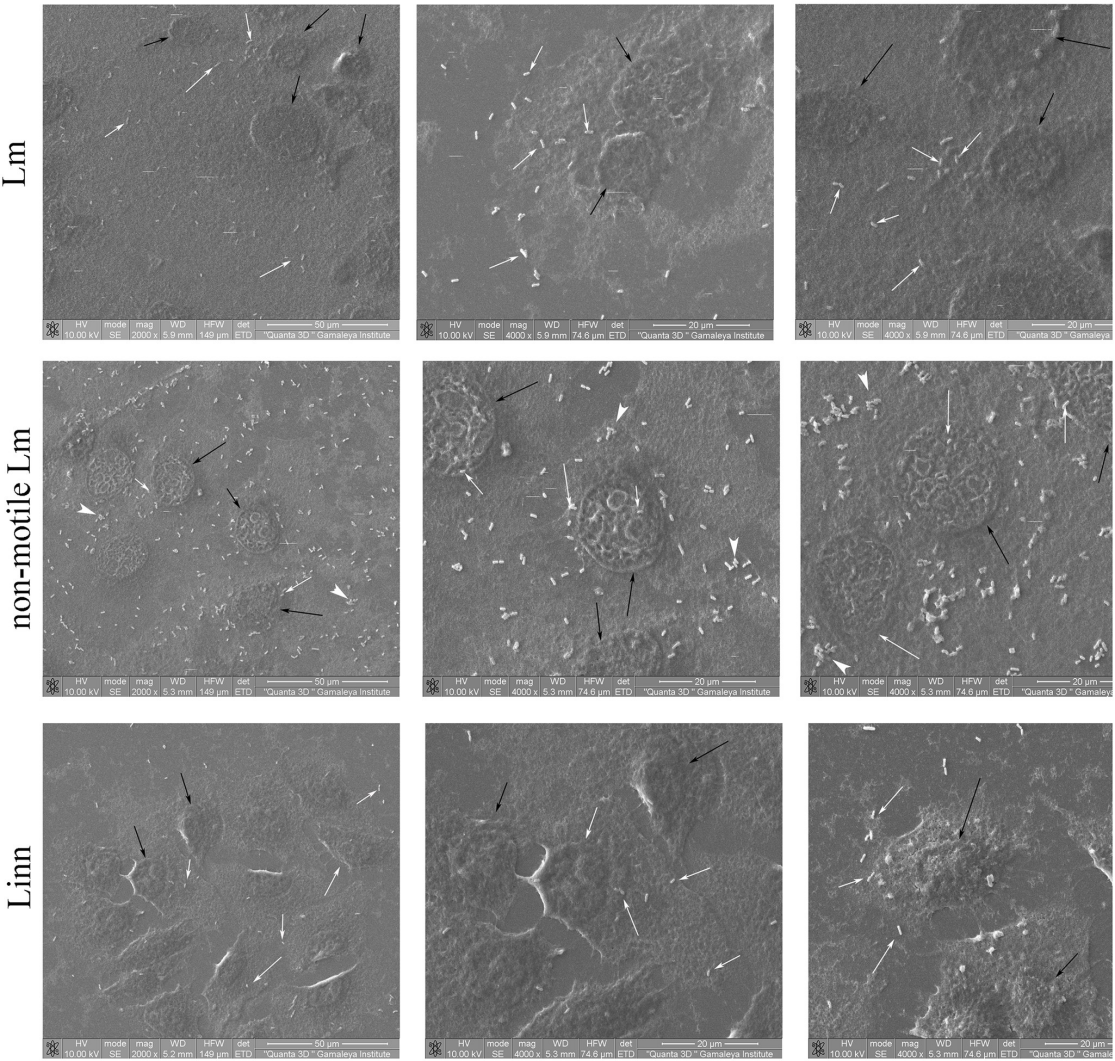
SEM analysis of adhesion to the surface of the HEp-2 cell surface. Black arrows–nuclei, while arrows–bacteria. White arrowheads–bacterial aggregates.

To get more clear evidences on bacterial distribution over cell surface, we used scanning electron microscopy (SEM). SEM supported a view of relatively uniform distribution of non-motile bacteria, which were often represented by small flocs over cell surface including areas over the cell nucleus (Fig 5). Motile bacteria of both pathogenic and saprophytic species were less numerous and their distribution was uneven with areas of increased bacterial concentration that located mainly at the cell periphery with the increasing number of bacteria toward the cell edge and the cell-cell border.

### Motility improved *L*. *monocytogenes* invasion into epithelial cells in the course of transition from the ambient temperature to the human body temperature

**Fig 6:**
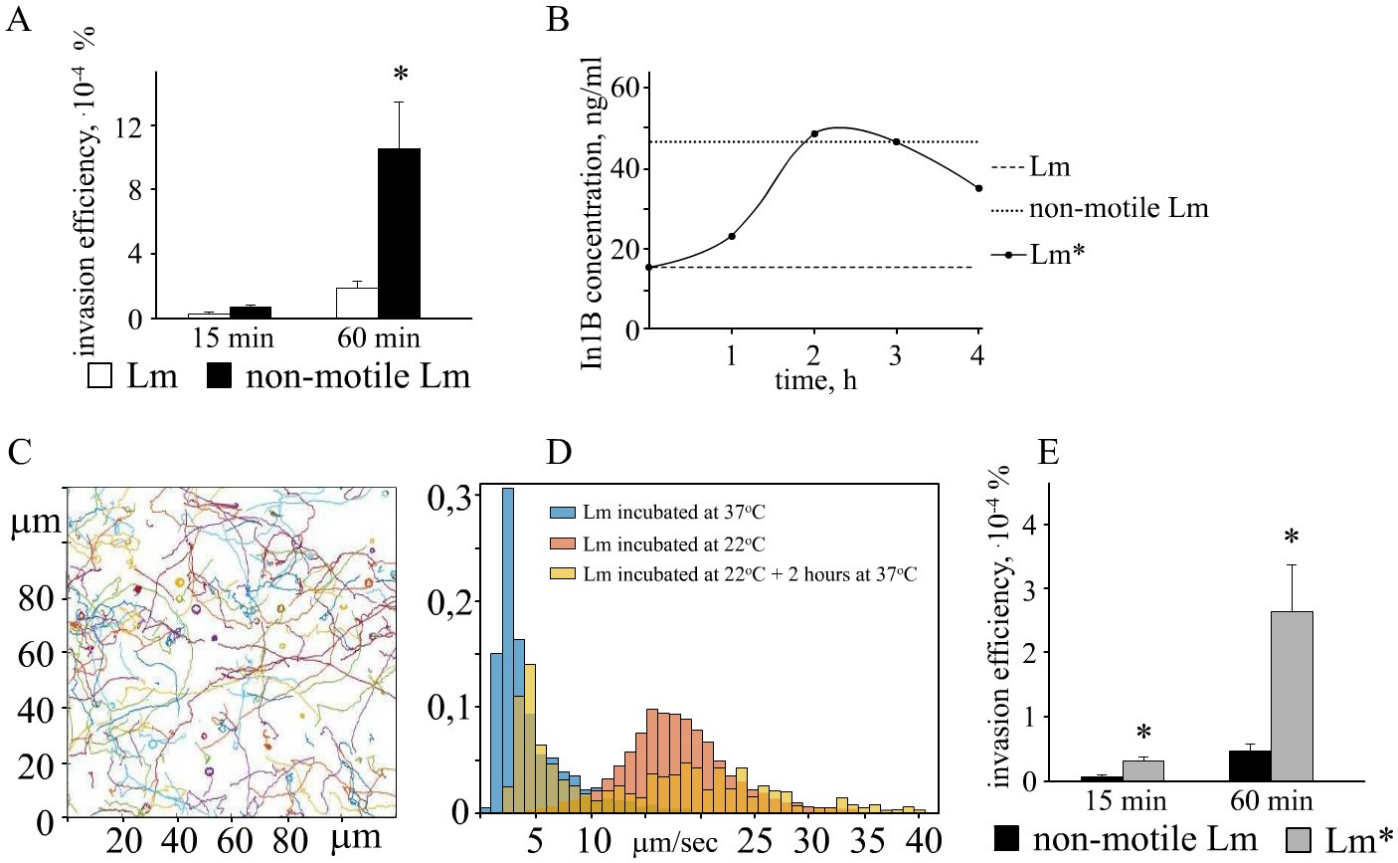
*L*. *monocytogenes* invasion efficiency into HEp-2 cells in dependence on motility and InlB production. (A)–invasion efficiencies of Lm and non-motile Lm, bacteria were co-incubated with HEp-2 cells 15 or 60 minutes before gentamicin was added. (B)–InlB production levels: InlB was detected on the bacterial surface using ELISA as described in Matetrials and Methods. (C)–individual trajectories of Lm* bacteria grown at 22°C and shifted to 37°C for two h; (D)–distribution of individual velocities for Lm (rose; grown at 22°C), non-motile Lm (blue, grown at 37°C), and Lm* (yellow; grown at 22°C followed by the 2h shift to 37°C) cultures; (E)—invasion efficiencies of Lm* and non-motile Lm, bacteria were co-incubated with HEp-2 cells 15 or 60 minutes before gentamicin was added. Lm–the culture was grown at 22°C overnight, bacteria were motile; InlB production was low; non-motile Lm–the culture was grown at 37°C overnight, bacteria were non-motile; InlB production was high; Lm*—the culture was grown at 22°C overnight and then shifted to 37°C, bacteria were motile; InlB production was high.

For *L*. *monocytogenes* as a facultative intracellular parasite, it is important that adhesion leads to cell invasion. Invasion of non-motile Lm and motile Lm into HEp-2 cells was compared using the gentamicin test. Gentamicin was added to the culture 15 and 60 minutes past bacterial addition to leave alive only intracellular bacteria. After 15-minute adhesion, the invasion efficiencies of non-motile Lm and motile Lm were low and similar (2.0 x 10^−5^% and 5.9 x 10^−5^%, respectively; Fig 6A). However, after 60-minutes adhesion, the invasion of non-motile bacteria was almost 10 times more effective than motile (0.001% and 0.0001% respectively; p<0.01).

We suggested that the low invasion efficiency of motile Lm could be due to repression of the virulence regulon in bacteria grown at the ambient temperature. To up-regulate the virulence regulon and particularly to increase expression of the invasion factors InlA and InlB required for active invasion into epithelial cells, we transfer the motile Lm culture to the temperature of 37°C, that is the temperature of the human body. Up-regulation of invasion factors at the culture transferred to the temperature of 37°C was monitored by accumulation of InlB at the bacterial surface. The shift of the motile Lm culture grown at 22°C to 37°C gave rise to InlB surface presentation in the amount comparable with non-motile Lm grown at 37°C overnight (Fig 6B). This effect was observed after 2 h and further incubation of motile Lm at did not improve InlB surface presentation.

The motility of bacteria transferred to 37°C for 2 h was studied as described above (Fig 6C) and compared with motility of the cultures grown at 22°C and 37°C (Fig 6D). The culture grown overnight at the ambient temperature and transferred to 37°C for 2 h was designated Lm*. The number of non-motile bacteria increased after the 2 h shift 37°C to reach 44% if compared with 12% non-motile bacteria in the 22°C Lm culture. Still, more than a half of Lm* bacteria were motile and the distribution of individual velocities was similar in Lm and Lm* cultures (Fig 6D).

We compared invasion efficiencies of non-motile Lm and motile Lm* in the gentamicin test. After 15-minute adhesion, invasion of motile Lm* was at least five times more effective than invasion of non-motile Lm (p<0.01; Fig 6E). After 60-minutes adhesion, invasion efficiency of motile Lm* and non-motile Lm differed 2.5 times (p<0.01) despite much higher number of non-motile bacteria adhered to the cell surface (see. Fig 4B). Thus, motility markedly improved the in vitro invasion of bacteria expressing invasion factors.

## Discussion

Here we characterized *Listeria* spp motility at the NSS stage, i.e. the stage when the bacterium moving close to the surface to have short physical contacts with it. Pathogenic *L*. *monocytogenes* (Lm) and its closest saprophytic species *L*. *innocua* (Linn) demonstrated similar characteristics of motility thus suggesting that motility patterns did not depend on virulence traits.

Following individual trajectories, we observed the anisotropy of the motion vector distribution (Fig 2B) that suggested a certain coordination of moving cells. This coordination could appear as a result of inelastic cell-cell collisions and transient motility of paired cells. Such pair-wise interactions of bacterial cells are not possible in the bulk where bacteria avoid each other but appear to be possible in the course of NSS near the surface [43, 44].

To reveal a role of the NSS motility type for further interactions of bacteria with the surface, we compared adherence patterns of motile and non-motile Listeria spp docking at the plastic surface or at the surface of epithelial HEp-2 cells. Motile bacteria showed a clear difference in adhesion patterns depending on the surface type. Both pathogenic and saprophytic *Listeria* spp adhered to the surface as single or double cells evenly distributed over the plastic surface but preferentially adhered at the HEp-2 cell periphery avoiding surface over the cell nucleus. The last result is in line with results obtained by Misselwitz and co-workers who demonstrated for another foodborne pathogens *Salmonella Typhimurium* that the NSS motility results in preferential binding at sites of prominent topology, i.e. the base of rounded-up cells and membrane ruffles [15]. Similar adhesion at topologically prominent sites we observed for the *E*. *coli* strain M17 [29]. However, *E*. *coli* M17 preferentially adhered as sites where physical obstacles were found independently whether these were plastic or cell surface, while motile Listeria were distributed evenly over the plastic surface. Besides physical obstacles, unevenness of *Listeria* distribution over the cell surface might be due to sensitivity to other surface properties such as nanoscale changes in the surface slip or interactions with surface receptors [45]. Still, similarity of adhesion patterns demonstrated by motile Lm and Linn evidences against receptor interactions at the stopping stage and suggested that only NSS characteristic but not virulence properties are responsible for the observed irregularity over cell surface.

Our results demonstrated that temperature-dependent up-regulation of the virulence regulon and particularly the invasion factor InlB is a key feature to give an advantage in cell invasion for motile *L*. *monocytogenes* over non-motile bacteria. When motile and non-motile *L*. *monocytogenes* expressed the invasion factor InlB at the same level, motile Lm* invaded host cells up to 8-times more efficiently comparatively with non-motile Lm. These results suggested that motility driven bacterial location at the cell periphery was important for further events required for *L*. *monocytogenes* active invasion into epithelial cells. Particularly, peripheral location might be important for interactions with cell surface receptors important for invasion that are located at the cell edge. Indeed, E-cadherin, which is cognitive receptor of the invasion factor InlA is an essential component of adherence junctions [46]. The InlB receptor c-Met is largely distributed in dynamic insoluble plasma membrane fractions, known as lipid rafts, which have a tendency to move to the cell periphery and tight junctions [47, 48]. Thus, obtained results suggested that motility-driven bacterial accumulation at the cell periphery is advantageous for the pathogenic *L*. *monocytogenes* as it can improve the receptor-dependent cell invasion.

The 2 h shift of the *L*. *monocytogenes* culture grown at the ambient temperature to the temperature of the human body was enough to improve invasion factor expression while bacterial motility maintained. This period is comparable with the time required for contaminated food to enter the intestine suggesting that the mechanisms involving upregulation of invasion factor together with the motility maintenance might be active in the human infection.

Taken together, obtained results demonstrated that the *Listeria* spp NSS motility does not depend on virulence traits. Similar motility and adhesion patterns of the pathogenic species *L*. *monocytogenes* and its saprophytic counterpart *L*. *innocua* excludes a role of virulence factors in the uneven bacterial distribution over cell surface suggesting that it is specific NSS motility that defined *Listeria* spp prevailed distribution at the cell periphery. Such a peripheral location improved *L*. *monocytogenes* invasion into human cells under conditions of temperature-dependent up-regulation of virulence factors. Obtained results suggested that motility-driven bacterial accumulation at the cell periphery can be advantage for pathogens, interacting with receptors located at apical junctions.

## Supporting information

S1 Fig Reconstructed bacterial trajectories, color-coded according to the magnitude of instantaneous velocities. Typical individual trajectories (right) and typical picture of overall reconstructed tracks in the field of view of the microscope (left).
(TIF)

S2 Fig Illustration of the image processing for quantification of bacterial positioning over the cell surface. Image processing includes the following steps: 1) determination of cell borders (black lines) and bacterial positions (red dots); 2) drawing of radial lines from the nominal cell center through the bacterium to the cell border (white lines); 3) the line lengths from the nucleus edge to the cell edge were divided into deciles and bacterial positions were prescribed to the particular decile. All bacteria over the cell nucleus were prescribed to the first decile.
(TIF)

## References

[1] Josenhans C, Suerbaum S. The role of motility as a virulence factor in bacteria. Int J Med Microbiol. 2002;291: 605–614. doi: 10.1078/1438-4221-00173 12008914

[2] Duan Q, Zhou M, Zhu L, Zhu G. Flagella and bacterial pathogenicity. J Basic Microbiol. 2013;53: 1–8. doi: 10.1002/jobm.201100335 22359233

[3] Chaban B, Hughes HV, Beeby M. The flagellum in bacterial pathogens: For motility and a whole lot more. Semin Cell Dev Biol. 2015;46: 91–103. doi: 10.1016/j.semcdb.2015.10.032 26541483

[4] Baban ST, Kuehne SA, Barketi-Klai A, Cartman ST, Kelly ML, Hardie KR, et al. The role of flagella in Clostridium difficile pathogenesis: comparison between a non-epidemic and an epidemic strain. PLoS One. 2013;8: e73026. doi: 10.1371/journal.pone.0073026 24086268 PMC3781105

[5] Rogers TJ, Paton JC, Wang H, Talbot UM, Paton AW. Reduced virulence of an fliC mutant of Shiga-toxigenic Escherichia coli O113:H21. Infect Immun. 2006;74: 1962–1966. doi: 10.1128/IAI.74.3.1962-1966.2006 16495575 PMC1418679

[6] Jia T, Liu B, Mu H, Qian C, Wang L, Li L, et al. A Novel Small RNA Promotes Motility and Virulence of Enterohemorrhagic Escherichia coli O157:H7 in Response to Ammonium. MBio. 2021;12: 1–19. doi: 10.1128/mBio.03605-20 33688013 PMC8092317

[7] O’Neil HS, Marquis H. Listeria monocytogenes flagella are used for motility, not as adhesins, to increase host cell invasion. Infect Immun. 2006;74: 6675–6681. doi: 10.1128/IAI.00886-06 16982842 PMC1698079

[8] Gu H. Role of Flagella in the Pathogenesis of Helicobacter pylori. Curr Microbiol. 2017;74: 863–869. doi: 10.1007/s00284-017-1256-4 28444418 PMC5447363

[9] Young GMG, Smith MJM, Minnich SAS, Miller VVL. The Yersinia enterocolitica motility master regulatory operon, flhDC, is required for flagellin production, swimming motility, and swarming motility. J Bacteriol. 1999;181: 2823–2833. doi: 10.1128/JB.181.9.2823-2833.1999 10217774 PMC93725

[10] Kamp HD, Higgins DE. A protein thermometer controls temperature-dependent transcription of flagellar motility genes in Listeria monocytogenes. PLoS Pathog. 2011;7: e1002153. doi: 10.1371/journal.ppat.1002153 21829361 PMC3150276

[11] Minnich SA, Rohde HN. A rationale for repression and/or loss of motility by pathogenic Yersinia in the mammalian host. Adv Exp Med Biol. 2007;603: 298–311. doi: 10.1007/978-0-387-72124-8_27 17966426

[12] Mitchell G, Kogure K, Mitchell JG, Kogure K. Bacterial motility: links to the environment and a driving force for microbial physics. FEMS Microbiol Ecol. 2006;55: 3–16. doi: 10.1111/j.1574-6941.2005.00003.x 16420610

[13] Grognot M, Taute KM. More than propellers: how flagella shape bacterial motility behaviors. Curr Opin Microbiol. 2021;61: 73–81. doi: 10.1016/j.mib.2021.02.005 33845324

[14] Subramanian S, Kearns DB. Functional regulators of bacterial flagella. Annu Rev Microbiol. 2019;73: 225. doi: 10.1146/annurev-micro-020518-115725 31136265 PMC7110939

[15] Misselwitz B, Barrett N, Kreibich S, Vonaesch P, Andritschke D, Rout S, et al. Near surface swimming of Salmonella Typhimurium explains target-site selection and cooperative invasion. Plos Pathog. 2012;8: e1002810. doi: 10.1371/journal.ppat.1002810 22911370 PMC3406100

[16] Bianchi S, Saglimbeni F, Di Leonardo R. Holographic Imaging Reveals the Mechanism of Wall Entrapment in Swimming Bacteria. Phys Rev. 2017; 7: 011010 doi: 10.1103/PhysRevX.7.011010

[17] Chawla R, Gupta R, Lele TP, Lele PP. A Skeptic’s Guide to Bacterial Mechanosensing. J Mol Biol. 2020;432: 523–533. doi: 10.1016/j.jmb.2019.09.004 31629771 PMC7002054

[18] Antani JD, Gupta R, Lee AH, Rhee KY, Manson MD, Lele PP. Mechanosensitive recruitment of stator units promotes binding of the response regulator CheY-P to the flagellar motor. Nat Commun. 2021;12: 5442. doi: 10.1038/s41467-021-25774-2 34521846 PMC8440544

[19] Jarrell KF, McBride MJ. The surprisingly diverse ways that prokaryotes move. Nat Rev Microbiol 2008 66. 2008;6: 466–476. doi: 10.1038/nrmicro1900 18461074

[20] Berg HC. The rotary motor of bacterial flagella. Annu Rev Biochem. 2003;72: 19–54. doi: 10.1146/annurev.biochem.72.121801.161737 12500982

[21] Minamino T, Kinoshita M, Namba K. Directional Switching Mechanism of the Bacterial Flagellar Motor. Comput Struct Biotechnol J. 2019;17: 1075–1081. doi: 10.1016/j.csbj.2019.07.020 31452860 PMC6700473

[22] Kearns DB. A field guide to bacterial swarming motility. Nat Rev Microbiol. 2010;8: 634–644. doi: 10.1038/nrmicro2405 20694026 PMC3135019

[23] Berg HC, Anderson RA. Bacteria swim by rotating their flagellar filaments. Nature. 1973;245: 380–382. doi: 10.1038/245380a0 4593496

[24] Altindal T, Xie L, Wu XL. Implications of three-step swimming patterns in bacterial chemotaxis. Biophys J. 2011;100: 32–41. doi: 10.1016/j.bpj.2010.11.029 21190654 PMC3010836

[25] Taktikos J, Stark H, Zaburdaev V. How the motility pattern of bacteria affects their dispersal and chemotaxis. PLoS One. 2013;8: e81936. doi: 10.1371/journal.pone.0081936 24391710 PMC3876982

[26] Damton NC, Turner L, Rojevsky S, Berg HC. Dynamics of bacterial swarming. Biophys J. 2010;98: 2082–2090. doi: 10.1016/j.bpj.2010.01.053 20483315 PMC2872219

[27] Kaiser D. Bacterial swarming: a re-examination of cell-movement patterns. Curr Biol. 2007;17: R561–R570. doi: 10.1016/j.cub.2007.04.050 17637359

[28] Eisenstecken T, Hu J, Winkler RG. Bacterial swarmer cells in confinement: a mesoscale hydrodynamic simulation study Soft Matter. 2016;12: 8316. doi: 10.1039/c6sm01532h 27714355

[29] Abdulkadieva MM, Sysolyatina E V., Vasilieva E V., Gusarov AI, Domnin PA, Slonova DA, et al. Strain specific motility patterns and surface adhesion of virulent and probiotic Escherichia coli. Sci Rep. 2022;12: 614. doi: 10.1038/s41598-021-04592-y 35022453 PMC8755817

[30] Allerberger F, Wagner M. Listeriosis: a resurgent foodborne infection. Clin Microbiol Infect. 2010;16: 16–23. doi: 10.1111/j.1469-0691.2009.03109.x 20002687

[31] Cossart P, Pizarro-Cerdá J, Lecuit M. Invasion of mammalian cells by Listeria monocytogenes: Functional mimicry to subvert cellular functions. Trends in Cell Biology. 2003. 13: 23–31. doi: 10.1016/s0962-8924(02)00006-5 12480337

[32] Lingnau A, Domann E, Hudel M, Bock M, Nichterlein T, Wehland J, et al. Expression of the Listeria monocytogenes EGD inlA and inlB genes, whose products mediate bacterial entry into tissue culture cell lines, by PrfA-dependent and -independent mechanisms. Infect Immun. 1995;63: 3896–3903. doi: 10.1128/iai.63.10.3896-3903.1995 7558297 PMC173548

[33] Peel M, Donachie W, Shaw A. Temperature-dependent expression of flagella of Listeria monocytogenes studied by electron microscopy, SDS-PAGE and western blotting. J Gen Microbiol. 1988;134: 2171–2178. doi: 10.1099/00221287-134-8-2171 3150978

[34] Loh E, Dussurget O, Gripenland J, Vaitkevicius K, Tiensuu T, Mandin P, et al. A trans-acting riboswitch controls expression of the virulence regulator PrfA in Listeria monocytogenes. Cell. 2009;139: 770–779. doi: 10.1016/j.cell.2009.08.046 19914169

[35] Bigot A, Pagniez H, Botton E, Fréhel C, Dubail I, Jacquet C, et al. Role of FliF and FliI of Listeria monocytogenes in flagellar assembly and pathogenicity. Infect Immun. 2005;73: 5530–5539. doi: 10.1128/IAI.73.9.5530-5539.2005 16113269 PMC1231047

[36] Dons L, Eriksson E, Jin Y, Rottenberg MME, Kristensson K, Larsen CN, et al. Role of flagellin and the two-component CheA/CheY system of Listeria monocytogenes in host cell invasion and virulence. Infect Immun. 2004;72: 3237–3244. doi: 10.1128/IAI.72.6.3237-3244.2004 15155625 PMC415653

[37] Wortel IMN, Kim S, Liu AY, Ibarra EC, Miller MJ. Listeria motility increases the efficiency of epithelial invasion during intestinal infection. Knodler L, editor. PLoS Pathog. 2022;18: e1011028. doi: 10.1371/journal.ppat.1011028 36584235 PMC9836302

[38] Bagatella S, Tavares-Gomes L, Oevermann A. Listeria monocytogenes at the interface between ruminants and humans: A comparative pathology and pathogenesis review. Vet Pathol. 2022;59: 186–210. doi: 10.1177/03009858211052659 34856818

[39] Glaser P, Frangeul L, Buchrieser C, Rusniok C, Amend A, Baquero F, et al. Comparative genomics of Listeria species. Science. 2001;294: 849–52. doi: 10.1126/science.1063447 11679669

[40] Kathariou S, Kanenaka R, Allen RD, Fok AK, Mizumoto C. Repression of motility and flagellin production at 37 degrees C is stronger in Listeria monocytogenes than in the nonpathogenic species Listeria innocua. Can J Microbiol. 1995;41: 572–577. doi: 10.1139/m95-076 7641140

[41] Petrov OF, Boltnev RE, Vasiliev MM. Experimental evolution of active Brownian grains driven by quantum effects in superfluid helium. 2022;12: 1–14. Available: https://pubmed.ncbi.nlm.nih.gov/35413969/10.1038/s41598-022-09523-z PMC9005707 35413969

[42] Kalinin E V., Chalenko YM, Kezimana P, Stanishevskyi YM, Ermolaeva SA. Combination of growth conditions and InlB-specific dot-immunoassay for rapid detection of Listeria monocytogenes in raw milk. J Dairy Sci. 2023;106: 1638–1649. doi: 10.3168/jds.2022-21997 36710191

[43] Swiecicki JM, Sliusarenko O, Weibel DB. From swimming to swarming: Escherichia coli cell motility in two-dimensions. Integr Biol (Camb). 2013;5: 1490–1494. doi: 10.1039/c3ib40130h 24145500 PMC4222179

[44] Ishikawa T, Sekiya G, Imai Y, Yamaguchi T. Hydrodynamic interactions between two swimming bacteria. Biophys J. 2007;93: 2217–2225. doi: 10.1529/biophysj.107.110254 17496014 PMC1959529

[45] Hu J, Wysocki A, Winkler RG, Gompper G. Physical Sensing of Surface Properties by Microswimmers—Directing Bacterial Motion via Wall Slip. Sci Rep. 2015;5: 9586. doi: 10.1038/srep09586 25993019 PMC4438609

[46] Coopman P, Djiane A. Adherens Junction and E-Cadherin complex regulation by epithelial polarity. 2016; 73: 3535–3553. doi: 10.1007/s00018-016-2260-8 27151512 PMC11108514

[47] Duhon D, Bigelow RLH, Coleman DT, Steffan JJ, Yu C, Langston W, et al. The polyphenol epigallocatechin-3-gallate affects lipid rafts to block activation of the c-Met receptor in prostate cancer cells. Mol Carcinog. 2010;49: 739–749. doi: 10.1002/mc.20649 20623641

[48] Lu Y-C, Chen H-C. Involvement of lipid rafts in adhesion-induced activation of Met and EGFR. 2011;18: 78. doi: 10.1186/1423-0127-18-78 22032640 PMC3244112

