# Supplementary figures and images for: Motility provides specific adhesion patterns and improves Listeria monocytogenes invasion into human HEp-2 cells
Source: PLoS One. 2023 Aug 31;18(8):e0290842. doi: 10.1371/journal.pone.0290842 (PMC10470941; doi:10.1371/journal.pone.0290842)

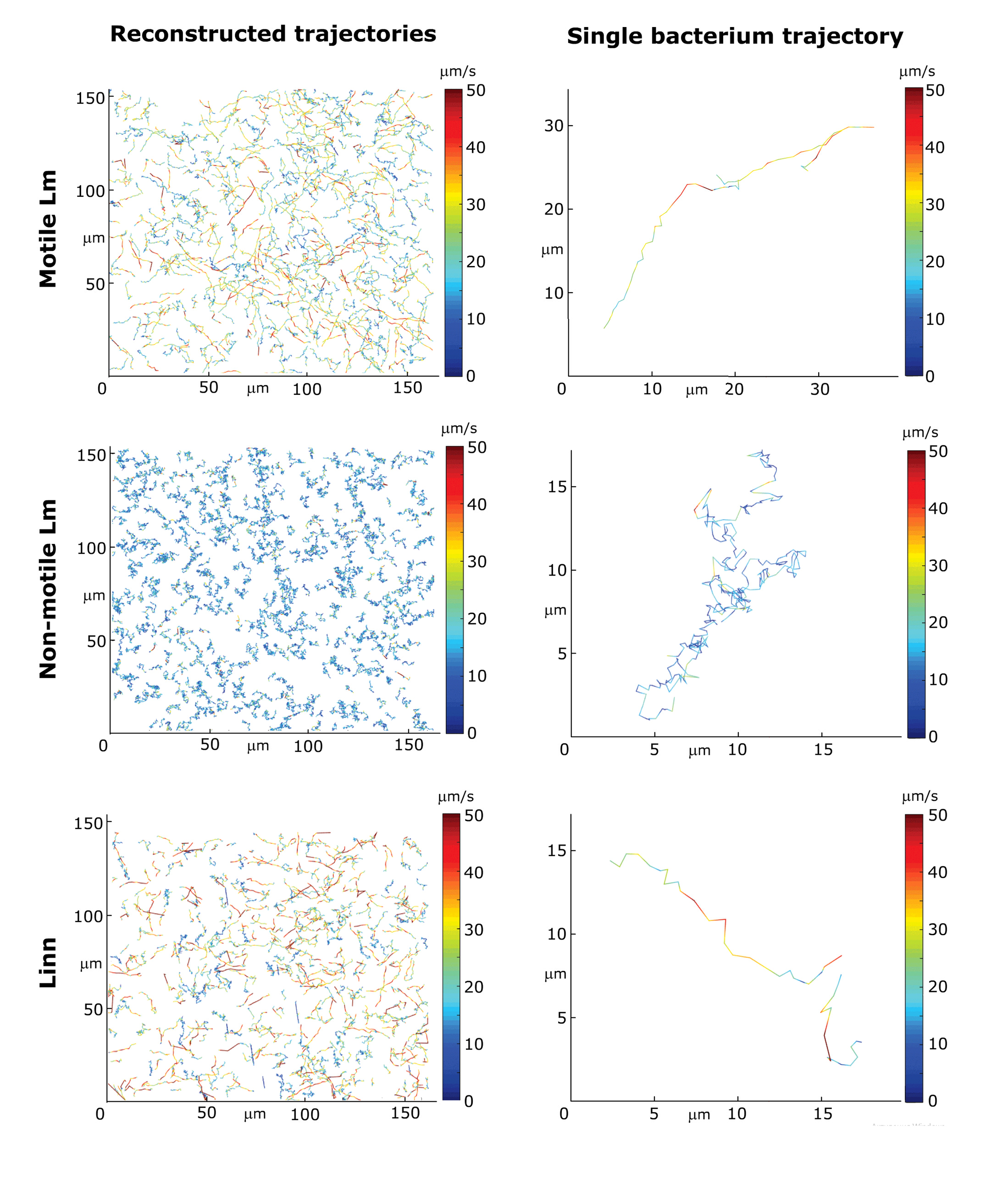

Supplement: S1 Fig — Typical individual trajectories (right) and typical picture of overall reconstructed tracks in the field of view of the microscope (left). (TIF) [file pone.0290842.s001.tif]

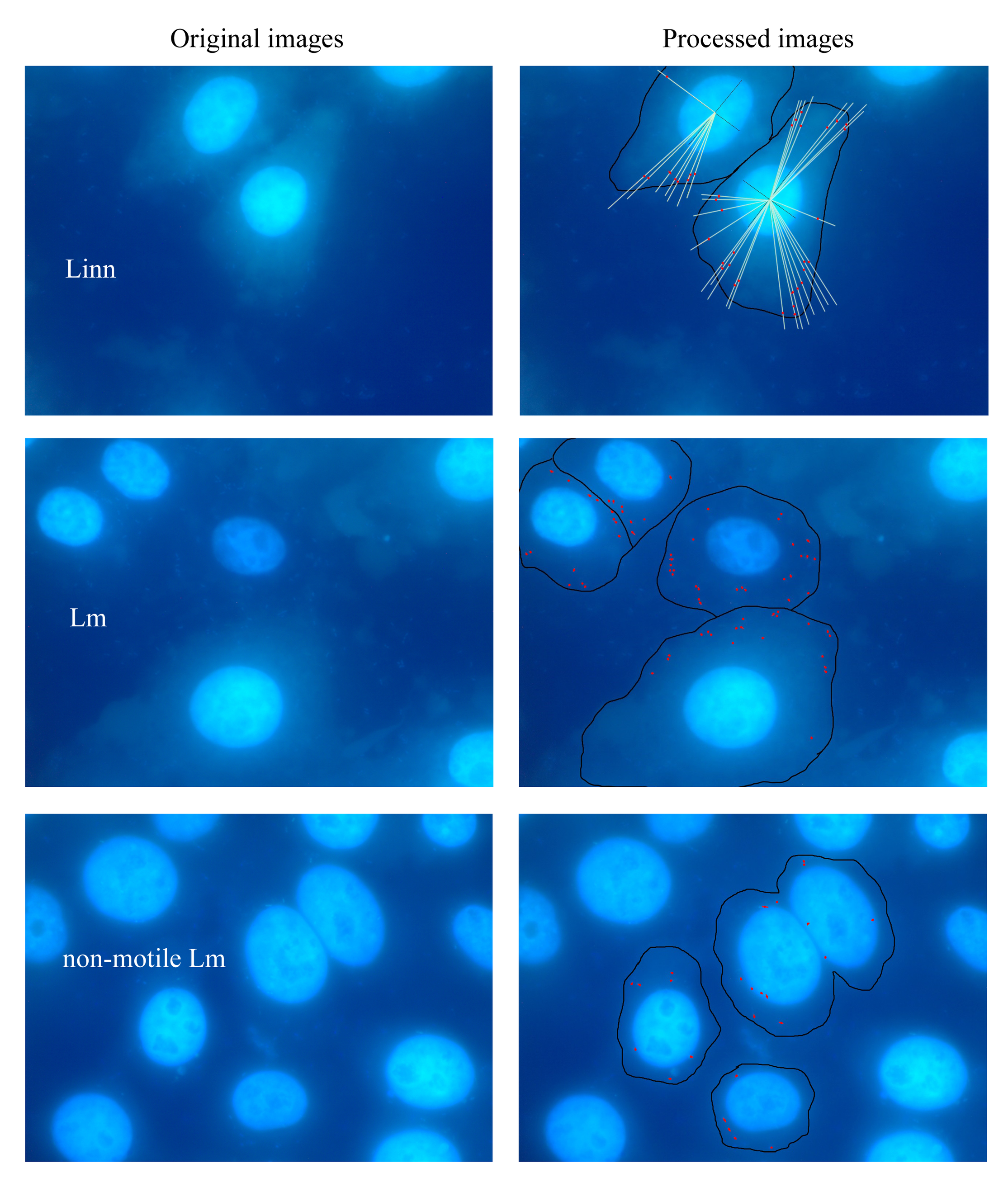

Supplement: S2 Fig — Image processing includes the following steps: 1) determination of cell borders (black lines) and bacterial positions (red dots); 2) drawing of radial lines from the nominal cell center through the bacterium to the cell border (white lines); 3) the line lengths from the nucleus edge to the cell edge were divided into deciles and bacterial positions were prescribed to the particular decile. All bacteria over the cell nucleus were prescribed to the first decile. (TIF) [file pone.0290842.s002.tif]
